# Interpreting the γ Statistic in Phylogenetic Diversification Rate Studies: A Rate Decrease Does Not Necessarily Indicate an Early Burst

**DOI:** 10.1371/journal.pone.0011781

**Published:** 2010-07-23

**Authors:** James A. Fordyce

**Affiliations:** Department of Ecology and Evolutionary Biology, University of Tennessee, Knoxville, Tennessee, United States of America; Field Museum of Natural History, United States of America

## Abstract

**Background:**

Phylogenetic hypotheses are increasingly being used to elucidate historical patterns of diversification rate-variation. Hypothesis testing is often conducted by comparing the observed vector of branching times to a null, pure-birth expectation. A popular method for inferring a decrease in speciation rate, which might suggest an early burst of diversification followed by a decrease in diversification rate is the γ statistic.

**Methodology:**

Using simulations under varying conditions, I examine the sensitivity of γ to the distribution of the most recent branching times. Using an exploratory data analysis tool for lineages through time plots, tree deviation, I identified trees with a significant γ statistic that do not appear to have the characteristic early accumulation of lineages consistent with an early, rapid rate of cladogenesis. I further investigated the sensitivity of the γ statistic to recent diversification by examining the consequences of failing to simulate the full time interval following the most recent cladogenic event. The power of γ to detect rate decrease at varying times was assessed for simulated trees with an initial high rate of diversification followed by a relatively low rate.

**Conclusions:**

The γ statistic is extraordinarily sensitive to recent diversification rates, and does not necessarily detect early bursts of diversification. This was true for trees of various sizes and completeness of taxon sampling. The γ statistic had greater power to detect recent diversification rate decreases compared to early bursts of diversification. Caution should be exercised when interpreting the γ statistic as an indication of early, rapid diversification.

## Introduction

The distribution, or timing, of cladogenic events in ultrametric trees provides an opportunity to test hypotheses regarding historical variation in diversification rates [1], [2], [3], [4], [5], [6]. Hypothesis testing and inference often involves comparing the observed tree, specifically the vector of branching times, to a null expectation, often a pure-birth, or Yule process, model of diversification [7], [8]. Under a pure-birth model of diversification, one with a constant rate and no extinction, a log-linear increase in the number of lineages is expected [7], [9]. Deviations from a log-linear increase can be used to reject a constant, pure-birth, model of diversification, and might be used to infer historical rate variation.

One commonly used method to detect deviations from a pure birth model is the constant-rates test proposed by Pybus and Harvey [7], often referred to simply by its test statistic, γ (equation 1 in [7]). The γ statistic has the appealing quality that under a pure-birth process, the distribution of γ follows a standard normal distribution. Thus, the hypothesis of a constant, pure-birth rate of diversification can be tested in a manner similar to a one-sample *t*-test. A deceleration in diversification rate is inferred when γ≤−1.645 (one-tailed test at α = 0.05). In this context, a deceleration in diversification rate implies an early burst of diversification, and this is the standard interpretation of a significant γ (e.g., [2], [4]). The constant-rates test has little power to detect acceleration in diversification rates because it cannot discriminate between acceleration and a constant rate with extinction [7]. Thus, its use has been largely restricted to inferring a decrease in speciation rate over the history of the tree, which might indicate an early burst of diversification or adaptive radiation early in the history of the tree. Incomplete lineage sampling can also affect the value of the γ statistic, here in a direction that favors the hypothesis of a decrease in diversification rate, interpreted as equivalent to an early burst. This is due to the fact that incomplete lineage sampling prunes tips from the tree, thus inflating the time interval between cladogenic events in the more recent past [9]. Pybus and Harvey [7] proposed a solution to this problem, the Monte Carlo constant rates test (MCCR test), where the critical value for rejecting a constant rate (the 0.05 quantile) is calculated by examining the distribution of γ for simulated trees that include the same incomplete lineage sampling supposed for the observed tree. A similar issue arises for how to treat the most recent time interval in the vector of branching times. For the most recent interval, we cannot know where the sampling time falls between the most recent cladogenic event and the next cladogenic event, because the later has yet to occur. A solution to this problem is to truncate the tree so that the most recent interval is excluded from the analysis [6], as this makes all intervals an outcome of the same random process. Accounting for incomplete lineage sampling via the MCCR test, and assuring that the vector of branching times are samples from the same process will insure the validity of statistically accepting or rejecting the null hypothesis. However, does a significant γ indicate an early burst of diversification?

Here, I examine whether detection of a decrease in diversification rates using γ necessarily requires that a high rate of diversification occurred near the base of a tree. I use an exploratory data analysis tool, tree deviation, to identify simulated trees that have, throughout their history, had a greater number of taxa than expected under a pure-birth model of diversification. Using simulations, I examine the sensitivity of γ to the most recent diversification rate history, and assess the power of γ to detect a shift towards decreased diversification rate.

## Results and Discussion

**Figure 1:**
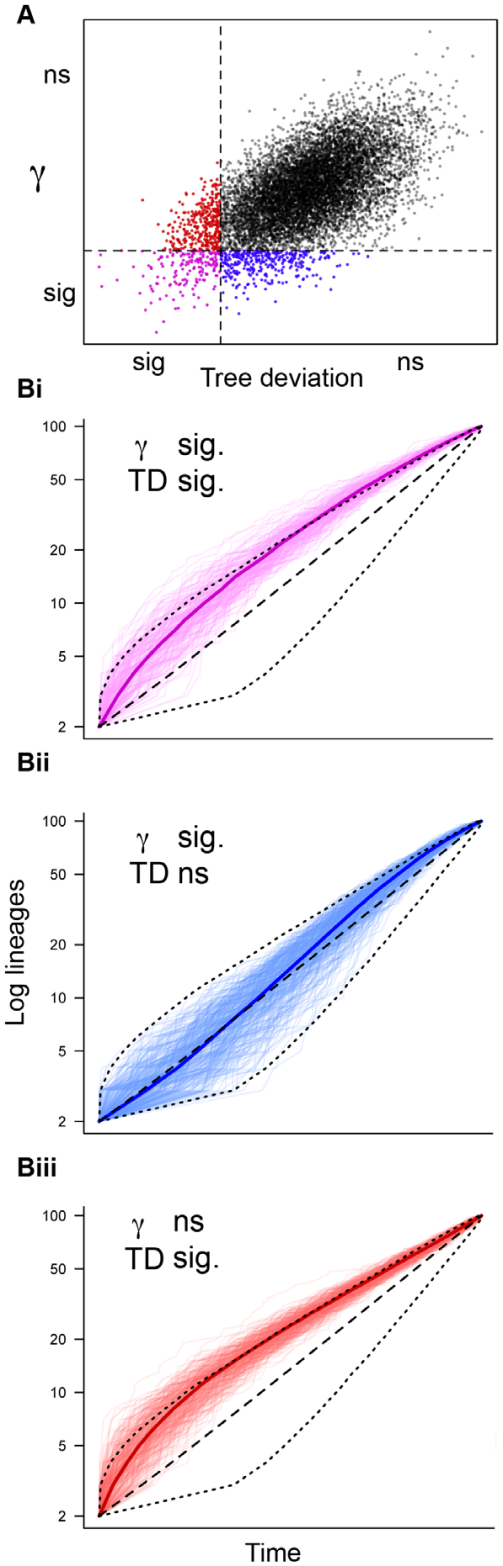
Distribution of γ and tree deviation (TD) values for 10000 simulated pure birth trees containing 100 taxa. A) γ and tree deviation values. Horizontal hatched line indicates 0.05 quantile for γ values (γ = −1.645). γ values below horizontal line are significant at α = 0.05. Vertical hatched line indicates 0.05 quantile for tree deviation. Values to left of vertical line can be considered significant at α = 0.05 (one-tailed). Purple indicates both γ and tree deviation significant, blue and red indicate only γ or tree deviation significant, respectively. B) Lineages through time plot for (i) γ and tree deviation significant (purple), (ii) only γ significant (blue), (iii) only tree deviation significant (red). Hatched lines indicate mean lineages through time plot and 0.025 and 0.975 quantiles for each subset of trees. Solid colored line indicates the mean lineages through time for the illustrated subset of trees.

The exploratory data analysis tool, tree deviation, was devised to detect trees that, on average throughout their history, have had a greater number of lineages than expected under a pure birth model (at α = 0.05). This is consistent with the expectation that following an early, high rate of diversification, without extinction, there will be more lineages. The tree deviation will not, however, detect deviation from a pure-birth expectation for trees with a more dynamic diversification rate history (e.g., a rapid burst, followed by an extreme decrease, followed by an extreme increase), nor will it necessarily be informative if extinction rates vary. Despite these limitations, values ≤0.05 quantile of tree deviation captured lineages with lineages through time plots that are consistent with the expectation of early, rapid diversification followed by a decrease in diversification rate (Figure 1). Agreement between significant γ and tree deviation ranged between 40% for 20 taxa trees to 20% for 100 taxa trees. Incomplete lineage sampling increased agreement between γ and tree deviation (52% agreement for 20 taxa trees at 50% taxon sampling to 28% agreement for 100 taxa trees at 50% taxon sampling). Figure 1A shows the agreement between γ and tree deviation for completely sampled 100 taxa trees. The region of agreement (i.e., both values below the 0.05 quantile) showed lineages through time plots that largely agree with the qualitative expectation for an early rapid diversification (Figure 1Bi). Where only γ was significant, there was no apparent trend consistent with early, rapid diversification (Figure 1Bii). Rather, an examination of the mean lineages through time shows a sigmoid shape, indicating a recent decrease in diversification rate without an early burst. Most striking is that trees identified by tree deviation that did not have significant γ values are qualitatively consistent with an early burst of diversification (Figure 1Biii).

**Figure 2:**
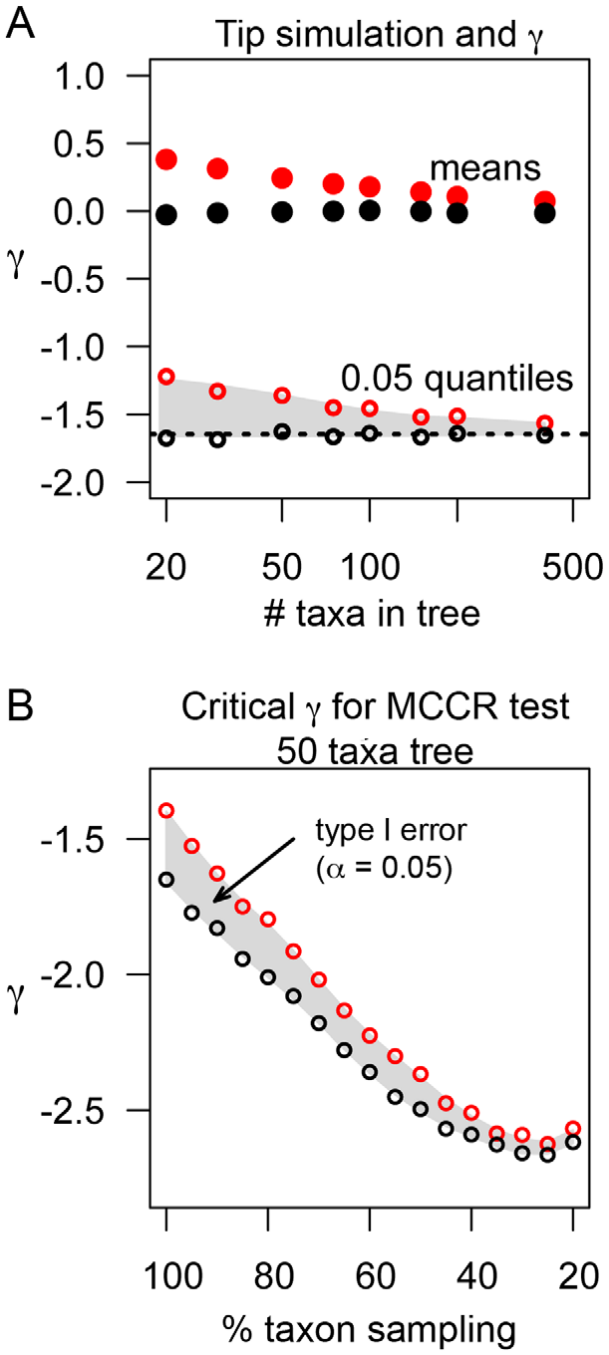
Sensitivity of γ to length of most recent terminal node. (A) Means (solid circles) and 0.05 quantiles (open circles) for 10000 simulated pure birth trees of various sizes. Black indicates entire most recent time interval simulated and red indicates zero time after the most recent cladogenic event. Hatched line indicates γ = −1.645 (critical threshold for significance of γ at α = 0.05). Shaded region shows discrepancy between 0.05 quantiles between simulation algorithms. (B) The persistent effect of most recent time interval simulation for trees simulated with incomplete lineage sampling. Shaded region shows discrepancy between 0.05 quantiles between simulation algorithms, where type I error at α = 0.05 occurs if simulation algorithm fails to simulate the entirety of most recent time interval. Open circle symbols same as above.

The means of γ for simulated trees that did not include the entirety of the final time interval were consistently greater than 0 across various tree sizes (i.e., number of taxa), whereas trees that included the entirety of the final interval agreed with the expected behavior of γ (Figure 2A). The discrepancy between simulation algorithms declined exponentially as the number of taxa in the simulated tree increased. This discrepancy remained for trees simulated with incomplete lineage sampling (Figure 2B), the procedure used for the MCCR test [7]. Thus, failure of a simulation algorithm used for the MCCR test to include the entirety of the final time interval will increase type I error rate. When the final time interval is truncated from the vector of branching times, the expected statistical properties of γ return, with a mean of 0 and variance of 1. This supports Weir's suggestion that the most recent node be truncated from trees constructed with empirical data [6]. However, it also demonstrates how extraordinarily sensitive γ is to the tips of a tree, as the only deviation from a pure-birth model in these simulations was the length of the most recent time interval.

**Figure 3:**
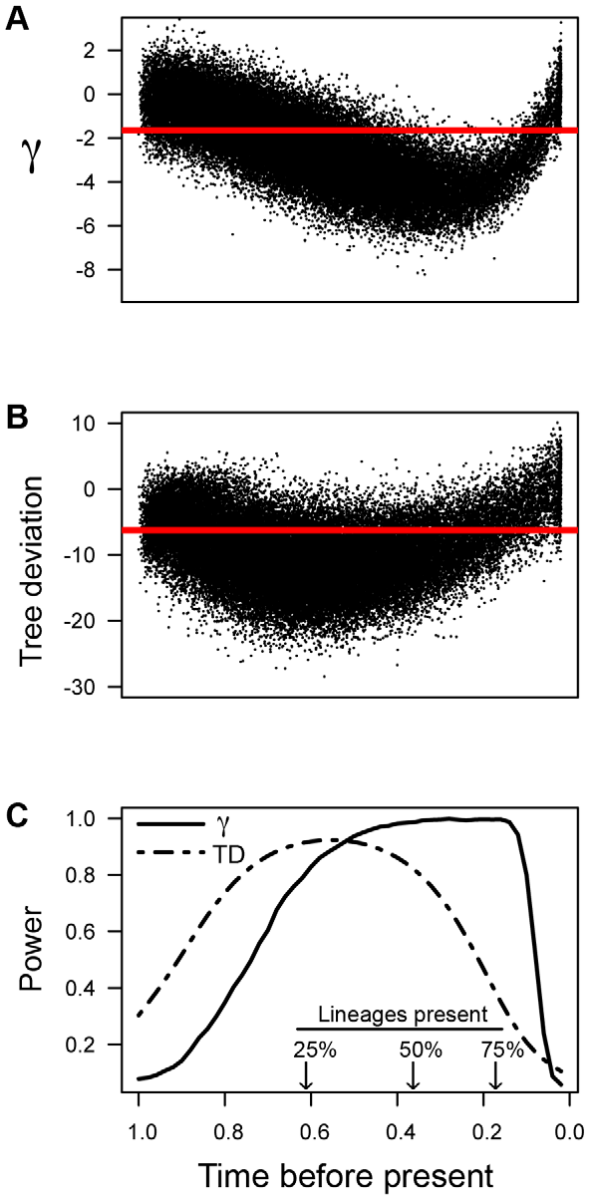
The power of γ to detect early bursts of diversification. (A) γ values for 50000 simulated trees containing 100 taxa with two diversification rates (a relatively high rate, followed by a lower rate). All trees scaled at 1 time unit total age. Time before present indicates the time of rate shift. Red horizontal line indicates significant γ at α = 0.05 (γ = −1.645). (B) Values for tree deviation (TD) across shift times for same 50000 trees. Red horizontal line indicates 0.05 quantile for 10000 pure birth trees containing 100 taxa scaled at 1 time unit total age. (C) Power (at α = 0.05) of γ and tree deviation (TD) to detect slow-down in diversification rates across possible shift-times for the 50000 simulated trees. Inset shows the median percent of total lineages present at a given time.

To examine the power of γ to detect a rate decrease, trees were simulated where an initial high rate of diversification was followed by a shift to a lower rate. Examination of 50000 simulated trees with varying rate shift-times showed that γ had increased sensitivity (i.e., significant at α = 0.05) to rate shifts that occurred in the more recent past, compared to rate shifts that occurred early in the history of the tree. The majority of trees with a rate shift occurring very early in their history were not significant (Figure 3A). The exploratory data analysis tool, tree deviation, similarly failed to consistently detect very early rate-shifts, though it was not nearly as sensitive to recent rate shifts as was the γ statistic (Figure 3B). Overall, γ had less power (at α = 0.05) compared to tree deviation to detect rate shifts in these simulations when the shift occurred early in a tree history (Figure 3C). The power of γ to detect a rate decrease was highest after 50% of the lineages were present, at a median time when 62% of the history of the tree had been simulated (Figure 3C).

The γ statistic has been used as an objective way to infer a decrease in diversification rate. A significant γ is often interpreted as evidence for an early, rapid burst of diversification. That is, a relatively high, early rate of diversification followed by a decrease in speciation rate. The ease in which γ can be calculated using various software packages, and its familiar statistical qualities, undoubtedly has facilitated its popularity. However, based on the simulations presented here, it is unclear whether the statistical hypothesis tested via the γ statistic is congruent with the biological hypothesis of early, rapid diversification. The γ statistic certainly detects a rate decrease under some scenarios; however, a recent rate decrease does not necessarily imply an early rate of cladogenesis. The γ statistic does, however, provide a powerful method to validate pure-birth tree simulation algorithms. Researchers should exercise caution when interpreting the γ statistic in studies examining diversification rate variation, and should supplement this approach with other methods aimed at detecting rate variation (e.g., [5], [8], [10]).

## Materials and Methods

Simulations and analyses were conducted in R [11], using functions from the APE [12], GEIGER [13], and LASER [14] packages, and the program Phylogen v1.1 [15]. The intervals between cladogenic events for a pure-birth tree can be simulated by random sampling from an exponential distribution, with the rate increasing as a function of the number of lineages present at a given time. On average, the resulting vector of branching times should describe a log-linear increase in taxa over time, with a mean γ of 0 with a variance of 1 [7].

### Tree deviation

**Figure 4:**
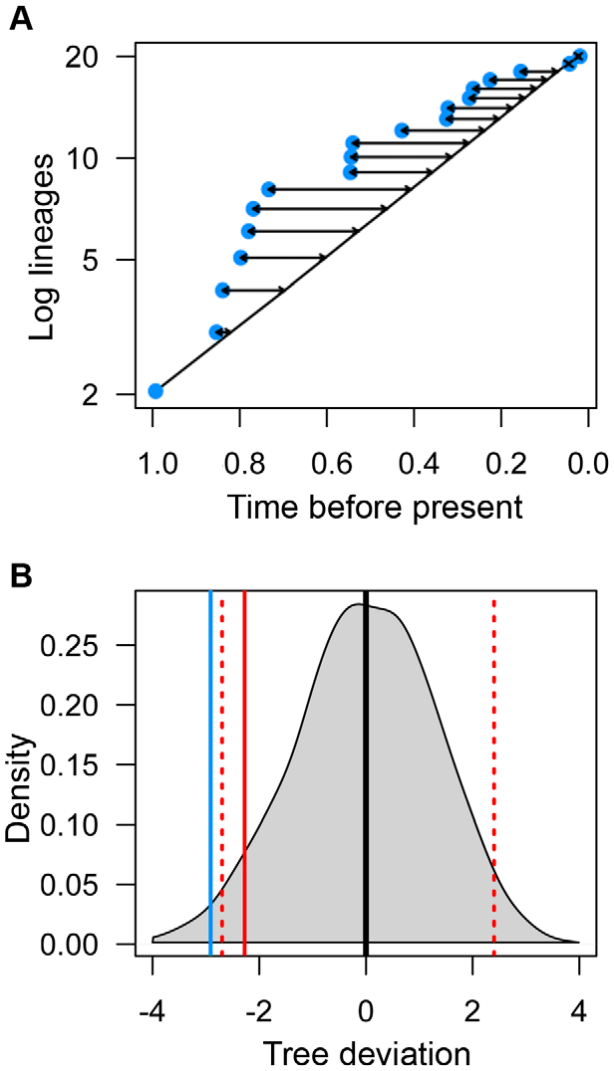
Calculation of tree deviation. (A) Blue dots indicate accumulation of lineages through time of focal tree. Black line indicates mean lineages through time for 50000 simulated pure birth trees containing 20 taxa. Arrows indicate the deviation of focal tree from simulated mean. (B) Density plot of tree deviation values from simulated trees. Black line indicates median and red hatched lines indicate 0.025 and 0.975 quantiles. Solid red line indicates 0.05 quantile used for one-tailed test. Blue line indicates deviation of focal tree.

Tree deviation was devised as an exploratory data analysis tool to examine the deviation in number of taxa across time of a given tree from the mean number of taxa expected under a pure-birth null expectation:
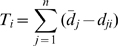
(1)Where *T_i_* is the deviation of the *i*th tree containing *n* nodes. The mean of *d_j_* is the mean time that *j* nodes are present based upon simulated pure-birth trees. *d_ji_* is the time that there are *j* nodes for the *i*th tree (Figure 4A). The distribution of tree deviations (TD) based on simulated pure-birth trees was used to identify trees that occurred below the 0.05 quantile, as these trees tend to be those with early, rapid accumulation of lineages (Figure 4B). A null distribution of tree deviations might be used to examine whether an empirically derived tree has had an excess of lineages throughout most if its history compared to a null, pure-birth expectation. Critical tree deviation values for “significance” can be calculated from the null distribution. Null trees can have incomplete lineage sampling, as in the MCCR test [7]. However, the power and behavior of the tree deviation method has not been explored under various histories of diversification. Thus, if employed, is best used in conjunction with other methods or as an exploratory data analysis tool.

The null tree deviation distribution was calculated using the following R code; where D is an array of branch times for ordered nodes (columns) of simulated trees of the same age (rows), STD is a vector containing deviation of nodes of a focal tree from the overall mean of simulated trees, and NTD is a vector of tree deviation scores of all trees. The 0.05 quantile of NTD was used to determine the critical threshold for identifying trees with an excess of early lineages (α = 0.05).

# Calculate tree deviation

STD<-NA

NTD<-NA

for (i in 1:length(D[,1])){

 for (j in 1:length(D[1,])){

  STD[j]<-mean(D[,j])-D[i,j]

  }

 NTD[i]<-sum(STD)

 }

### Simulation 1

10000 pure-birth trees containing 20, 30, 40, 50, 60, 70, 80, 90, and 100 taxa were simulated using a modification (see below) of the birthdeath.tree function in GEIGER [13]. Tree deviation and γ were recorded for each set of simulations and lineages through time plots were examined to qualitatively access the performance of γ. Trees with γ and tree deviation values equal to, or below, the 0.05 quantile were identified as trees that were significantly different from the null expectation (α = 0.05, one-tailed test).

### Simulation 2

10000 pure-birth trees containing 20, 30, 50, 75, 100, 150, 200, and 400 taxa were simulated under two scenarios. In one scenario, the simulation stopped once the desired number of taxa was obtained. Thus, the branch-length joining the most recent pair of taxa was zero. This is the algorithm used for the birthdeath.tree function in GEIGER as of version 1.2–13. This function was modified to simulate the entirety of the final time interval by simulating *n*+1 taxa, then pruning the most recent taxon. Thus, all remaining nodes were generated under the same random process. The sensitivity of γ to the tips of the trees was examined by comparing the values of γ under both simulation algorithms. Next, the values of γ under both simulation algorithms were compared under conditions of incomplete taxon sampling (ranging from 95% to 20% taxon sampling). Taxa were randomly removed, as is the standard protocol when implementing the MCCR test [7]. These simulations served to further examine the sensitivity of γ to the tips of trees, and to examine how this sensitivity might affect type I error rates for the MCCR test when the null distribution is based on trees that fail to simulate the entirety of the final branch length.

### Simulation 3

To examine the power of γ to detect a known rate decrease, 50000 trees containing 100 taxa were simulated with an initial high rate followed by a slower rate using the program Phylogen v1.1 [15]. Pure-birth trees simulated in Phylogen include the entirety of the most recent branch length, resulting in the desired statistical properties for γ (mean = 0, σ^2^ = 1). Using the command *episodic*, pure-birth trees were simulated with birth = 3, followed by birth = 1 after a given number of taxa had been simulated. The number of taxa present when the shift occurred ranged between 5 and 100. All trees were standardized to be 1 time unit in length. The performance of γ and tree deviation was accessed to examine the power of both methods to detect the simulated decrease in diversification rates.
